# The perinatal mental health experiences of black immigrant mothers in the UK: A qualitative systematic review and thematic synthesis

**DOI:** 10.1371/journal.pone.0331547

**Published:** 2025-12-19

**Authors:** Funmilayo Fabiyi, Zoe Darwin, Rajeeb Kumar Sah, Amanda Firth

**Affiliations:** School of Human and Health Sciences, University of Huddersfield, Huddersfield, United Kingdom; University of Zurich, SWITZERLAND

## Abstract

**Introduction:**

Mental health difficulties during and after pregnancy are common worldwide. Women from minoritised ethnic communities are disproportionately affected, as are those affected by migration factors. Research typically treats ethnic minority groups as homogenous despite having potentially different needs and experiences. In the UK, Black women face considerable maternal mental health disparities, warranting further attention.

**Aim:**

To explore and understand the perinatal mental health (PMH) views and experiences of Black immigrant mothers in the UK, the support that was available to them and, perspectives on support received.

**Methods:**

MAG Online Library, CINAHL, MEDLINE, PsycINFO and Scopus electronic databases were systematically searched. And thematic synthesis was undertaken with all the studies which met the eligibility criteria.

**Findings:**

Four studies were retrieved, all focusing on postnatal depression. The synthesis generated three themes: i) perceived causes of PMH difficulties, ii) symptoms, signs and impact of PMH difficulties, iii) available support and coping means. Cultural beliefs permeated across all the themes.

**Conclusion:**

Black immigrant mothers in the UK face challenges such as lack of support, inadequate information, cultural perceptions, and insufficient help from family, community, and healthcare professionals. Further research is needed that extends beyond postnatal depression and includes parent-infant relationships.

## Introduction

Globally, mental health-related illnesses affect approximately one in five women in the perinatal period; from conception until one year postpartum [1], with depression being the most common condition [2]. In the UK, perinatal mental health care operates under a stepped-care model, whereby women with mild-moderate needs are supported within primary care – involving GPs, midwives, health visitors, and talking therapies – and women with moderate-severe and complex needs are referred to specialist perinatal mental health services that have multi-disciplinary teams [3,4]. These specialist secondary services have seen rapid investment and expansion in recognition of the high economic cost of perinatal mental health difficulties (£8.1 billion per annual cohort of births), mainly due to the long-lasting impact on the child [5] and maternal suicide being a leading cause of death in the UK [6]. However, inequalities in access remain [5], particular for those from minoritised ethnic backgrounds [7], reflecting barriers concerning stigma, lack of culturally appropriate services, and systemic gaps in care provision [8].

Indeed, racial and ethnic disparities in mental health access and outcomes are well-established internationally. Women from ethnic minority backgrounds are both at a higher risk of experiencing perinatal mental health difficulties and less likely to receive diagnoses and treatment [9]. Immigration (here, used to refer to residing in a country other than the country of birth) introduces an additional layer of vulnerability for women in the perinatal period. Immigrant women in the host country face a higher risk of poor pregnancy and childbirth outcomes with subsequent implications for their mental and physical health with increased morbidity and mortality in contrast to women born in the country [10,11]. This includes increased risk of postnatal depression [12], which is even greater for forced migrant women [13]. Immigrant mothers are at risk due to various stressors throughout their migration journey like language barriers, social isolation, and discrimination [14–16]. The loss of cultural, social, and material resources can also affect their well-being during the perinatal period [17]. Indeed, a meta-synthesis by [18] on the experiences of postnatal depression (PND) in immigrant mothers living in Western countries found that immigrant mothers are subjected to different stressors following childbirth, which increases vulnerability to postnatal depression. Also implicated are a lack of social networks and support, low levels of education and income, and discrimination [19].

Accompanying this heightened vulnerability, immigrant mothers can find it challenging to access maternity care due to legal barriers, navigating complex health and social care systems, and lack of trust [15], all of which may be relevant for accessing support relating to perinatal mental health. Additionally, the research identifies additional barriers relating to a lack of awareness of maternal mental health [13,20] and a lack of culturally acceptable services that meet the needs of the diverse population [21,22]. Critically, dominant approaches continue to treat women from minority ethnic backgrounds as a homogenous group [18,20], despite different needs, which risks failing to deliver culturally competent care adequately.

In the UK, Black women face the greatest inequalities in maternal health [23]. The intersection of mental health, ethnicity, and migration forms a complex backdrop against which Black immigrant mothers in the UK experience perinatal care. In the UK, Black African and Caribbean women are more vulnerable to mental health problems compared to White women [24] and have poorer access to appropriate maternal mental healthcare than women from White ethnic background [25–27]. Black women’s views and experiences are shaped by a variety of structural and cultural factors. Studies indicate that many Black immigrant women feel marginalised within the UK healthcare system, reporting feelings of invisibility, lack of empathy from practitioners, and a perception that their emotional needs are not taken seriously [7,8].

Edge’s seminal work [8,28] finds Black and Minority Ethnic (BME) women in the UK are disproportionately affected by perinatal mental health challenges while simultaneously facing barriers to accessing adequate care. These barriers include cultural stigma, language difficulties, and a lack of culturally competent services [8,28]. Although Edge’s work includes important insights into the effects of racism, cultural stigma, and systemic neglect, it does not explicitly differentiate between UK-born and immigrant Black mothers. Responding to this critical gap in the literature, this review specifically addresses the experiences and needs of Black mothers in the UK who migrated from other countries. The decision to limit this review to the UK is grounded in the need to understand the perinatal mental health experiences of Black immigrant mothers within a specific and complex national context. The UK is home to a significant and growing population of Black immigrants, many of whom originate from Africa and the Caribbean, bringing with them diverse cultural backgrounds, migration histories, and health beliefs [29].

The systematic review was undertaken to address the following research questions.

- What are the views and experiences of Black immigrant mothers in the UK concerning perinatal mental health?
- What are the various types of support, including family, community, and healthcare support, available for Black immigrant mothers in their perinatal period?
- What are the views and experiences of Black immigrant mothers regarding the support they receive for their mental health?

## Methodology

### Design

This qualitative evidence synthesis used systematic review methodology and reporting follows ENTREQ guidelines [30]

### Inclusion criteria

- Studies focusing on or exploring Black immigrant Mothers’ views and experiences of perinatal mental health in the UK. For the purpose of this research, Black immigrant mothers are defined as any mother who had themselves migrated to the UK regardless of the reason for migration, type of migration, recency and whether or not their babies were born in the UK. Eligibility was not restricted by perinatal mental health difficulties, regardless of whether there is a diagnosed condition or not
- Empirical research
- Qualitative studies or the qualitative part of mixed-method studies
- Studies published in English
- No date restriction.

### Exclusion criteria

- Non-peer-reviewed articles (to ensure methodological rigor, reduce bias, ensure quality control and maintain the reliability and scientific credibility of the synthesised evidence).
- Excluded if a mixed sample (or heterogenous group) and experience of Black immigrant mothers could not be identified separately.

### Search and selection strategy

A review strategy was developed using the PICo Model (Population, Phenomenon of Interest, and Context) to guide the systematic search (Table 1), where the population is Black immigrant mothers, the phenomenon of Interest is perinatal mental health, and the Context is the UK [31].

**Table 1:** Search strategy using PICo.

	Population				Phenomenon of Interest						Context
Key Terms	Black	AND	Immigrants	AND	Perinatal	AND	Mental Health	AND	Experiences	AND	UK
Synonyms	African, Caribbean Afro-Caribbean		Migrants, Refugees, asylum		Antenatal, Postnatal, Postpartum, Perinatal, Maternity, Pregnancy*		Depression, distress, Anxiety, Emotion, Wellbeing, Psychology		View, Perceptions Attitude		United Kingdom, Scotland, England, Northern Ireland, Wales.

The Boolean operators (AND/OR) were used to ensure rigorous search terms. Multiple databases were searched, including the literature search was conducted in MAG Online Library, CINAHL, MEDLINE, PsycINFO and Scopus databases using a search strategy developed by combining relevant key terms (see S1 Table). Additionally, citation chaining [32] was used to ensure all relevant literature was accounted for. The search was not restricted by date due to the limited available research.

**Fig 1:**
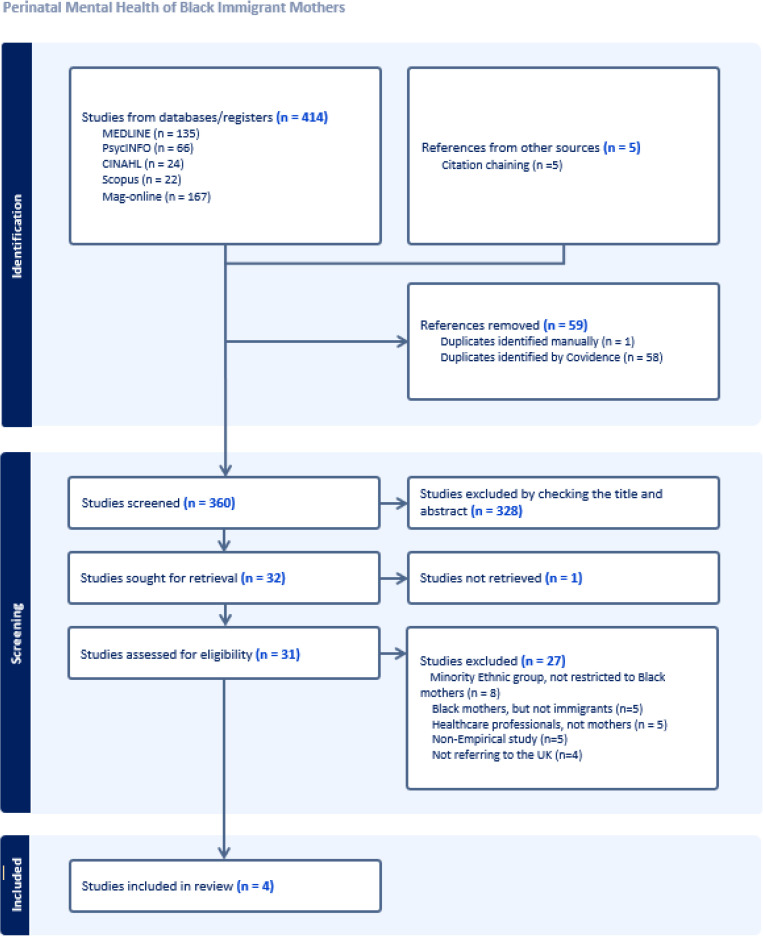
PRISMA diagram.

Two reviewers (FF and ZD) used Covidence screening software to independently screen and select studies using pre-determined eligibility criteria, initially based on the title and abstract, followed by a full-text review. Any disagreements were resolved in discussions with the other authors (RKS and AF) before making final decision. The PRISMA flow diagram [33] (Fig 1) provides further details, including reasons for exclusion.

PRISMA/PROSPERO: The researchers followed PRISMA guidance. The review protocol was registered on PROSPERO on 20/04/2023 (CRD42023406839 https://www.crd.york.ac.uk/prospero/display_record.php?ID=CRD42023406839).

### Quality appraisal

The Critical Appraisal Skills Programmes (CASP) Qualitative Checklist [34] was used to assess the quality of included papers (Table 2). The quality of each study was evaluated by one reviewer and corroborated by a second, with further discussion where required. All studies were found to be of good quality. The only weakness was the reflexivity of two of the included articles due to limited information regarding the relationship between the researchers and the participants. As part of the quality appraisal, we confirmed that all included studies had obtained appropriate ethical approval and employed suitable data analysis.

**Table 2:** Quality appraisal using the Critical Appraisal Skills Programme (CASP).

Qualitative Checklist Tool											
No	Author	Critical Appraisal Questions									
		1	2	3	4	5	6	7	8	9	10
1	Ling et al.	Yes	Yes	Yes	Yes	Yes	Yes	Yes	Yes	Yes	Yes
2	Dei-Anane et al.	Yes	Yes	Yes	Yes	Yes	**No**	Yes	Yes	Yes	Yes
3	Gardner et al.	Yes	Yes	Yes	Yes	Yes	**No**	Yes	Yes	Yes	Yes
4	Babatunde and Moreno-Leguizamon	Yes	Yes	Yes	Yes	Yes	Yes	Yes	Yes	Yes	Yes
The questions. 1. Was there a clear statement of the aims of the research? 2. Is a qualitative methodology appropriate? 3. Was the research design appropriate to address the aims of the research? 4. Was the recruitment strategy appropriate to the aims of the research? 5. Was the data collected in a way that addressed the research issue? 6. Has the relationship between researcher and participants been adequately considered? 7. Have ethical issues been taken into consideration? 8. Was the data analysis sufficiently rigorous? 9. Is there a clear statement of findings? 10. How valuable is the research? Valuable- Yes, not valuable -No											

### Data extraction and analysis

The review process followed a thematic synthesis framework adapted from Thomas and Harden [35] with three stages: coding findings, organising them into descriptive themes, and generating analytical themes. All texts within the finding sections were extracted. The study findings were read thoroughly and quotes from participants were coded. Descriptive themes were generated and analytical themes were subsequently generated, with consideration of the research questions [35]. Analysis and interpretation were discussed and refined across a series of meetings with all the four authors of the study, and a consolidated interpretation was developed.

The reviewers assessed the credibility of research findings using the Confidence in the Evidence from Reviews of Qualitative Research (CERQual) approach [36]. This method involves evaluating various factors such as the limitations of individual studies (methodology), relevance to the review question, coherence, and adequacy of data. The confidence in the evidence, which signifies the likelihood that the findings are reasonable representations of the phenomenon of interest, ranges from moderate to high, indicating a strong basis for the conclusions made. See Supporting Information (S2 and S3 Tables) for details.

## Results

A total of four studies were included in this research after an extensive review process involving the identification of 419 papers, which comprised 414 from various databases and 5 sourced through citation chaining, resulting in 360 unique records that were assessed for eligibility (see Fig 1). Collectively, these four studies encompassed a total of 54 participants, as detailed in Table 3.

**Table 3:** Summary of characteristics of included studies.

NO	Author and Year	Study Aims and Objectives	Study Setting	Study Design	Population and Sample Size	Data collection	Findings	Conclusion
1.	Ling et al. [39]	(1) To explore how first-generation Nigerian mothers in the UK experience postnatal depression; (2) To investigate how this group of mothers experience available treatment and resources, and how they manage and cope with Postnatal depression (PND).	UK (exact location not stated)	Qualitative. Interpretative phenomenological analysis (IPA)	6 Nigerian-born mothers.	Semi-structured interview.	The study gives evidence of the difficulties encountered by first-generation Nigerian Mothers in relation to PND (sociocultural factors, neglect from family and healthcare professionals, and self-reliance as a coping strategy).	The results show that in order to effectively involve mothers at an early stage, barriers impacting access to services must be addressed by promoting a patient-centred and culturally sensitive approach in healthcare teams.
2.	Dei-Anane et al. [37]	To examine the perception of Ghanaian migrant mothers living in London towards postnatal depression during the postnatal period.	UK London	Qualitative (Thematic Analysis)	25 Ghanaian mothers	In-depth interviews, augmented with informal conversations.	The study discovered that although Ghanaian migrant mothers reported going through stressful times, they hesitated to seek maternal mental health services assistance because they did not trust the healthcare providers they contacted. They appreciate the care of the health visitors, but the lack of family support worsened their stressful circumstances. They primarily turned to their religious leaders, acquaintances, and distant relatives for support when depressed.	Health professionals must make clear to mothers what their responsibilities are and take steps to evaluate migrant mothers on all factors that affect their postpartum experiences. The need for these mothers’ additional support needs to be identified by health experts is equally critical.
3.	Gardner et al. [38]	To explore the lived experience of postnatal depression (PND) in West African mothers living in the United Kingdom (UK).	UK. Manchester.	Qualitative (Interpretative Phenomenological Analysis)	6 West African Mothers (3 Nigerian 3 Ghanian)	Semi-structure interview	PND symptoms were present in women, but they did not see it as a disease. They believed that social stress was the cause of postnatal sadness and unhappiness. Participants claimed that their cultural background made it difficult to express depressive feelings, which negatively impacted their actions while seeking help.	The developed themes provide insight into the experiences of West African women living in England and add to the body of research on PND in Black and ethnic minority groups. These perceptions are essential for providing efficient, culturally appropriate treatment.
4.	Babatunde and Moreno-Leguizamon [40]	(1) To establish cultural elements related to postnatal depression through women’s narratives regarding their daily life situations, including the nuances and complexities present in postnatal depression. (2) To help health professionals understand and acknowledge postnatal depression signs in these immigrant women and some of the cultural ambiguities surrounding them.	UK South East London	Qualitative (Thematic analysis)	17 African Mothers (Nigeria, Ghana, Kenya, Somalia and Sierra Leone)	Focus group	Nearly half of the study participants deal with some symptoms of postnatal depression. The mothers did not see the symptoms as indicators of illness but rather as something else in their daily lives. The study also draws attention to the fact that despite spending a lot of time with the women, health visitors failed to notice the warning signs because intercultural communication, household and family politics, and women’s silence about their emotional struggles were not taken into account by the health services.	Health practitioners’ ability to comprehend any family’s dynamics was deemed crucial. It is crucial that the bio-psychosocial model of health services incorporates family politics. Health practitioners need to be aware of other presumed normalities outside the reconstructed British nuclear family in a multicultural community.

### Study characteristics

The four included studies were published between 2012–2022 (Table 3). All were qualitative studies but used varied approaches. While Dei-Anane et al. [37] used in-depth interviews, Gardner et al. [38] and Ling et al. [39] used semi-structured interviews for data collection, Babatunde and Moreno-Leguizamon [40] used a focus group discussion. Ling et al. [39] and Gardner et al. [38] used interpretive phenomenological analysis, and the other two used thematic analysis. The sample sizes ranged from six to 25 participants, reflecting the different approaches used. The participants in the studies were all from African countries, with two focused on West Africa, specifically Ghana and Nigeria [37–39], and one unrestricted, although with the majority (13/17) from Nigeria and Ghana [40]. All studies were focused on PND, with two concerning lived experiences [38,39] and two concerning perspectives in mothers, without the requirement of having direct own experience [37,40].

### Themes

**Fig 2:**
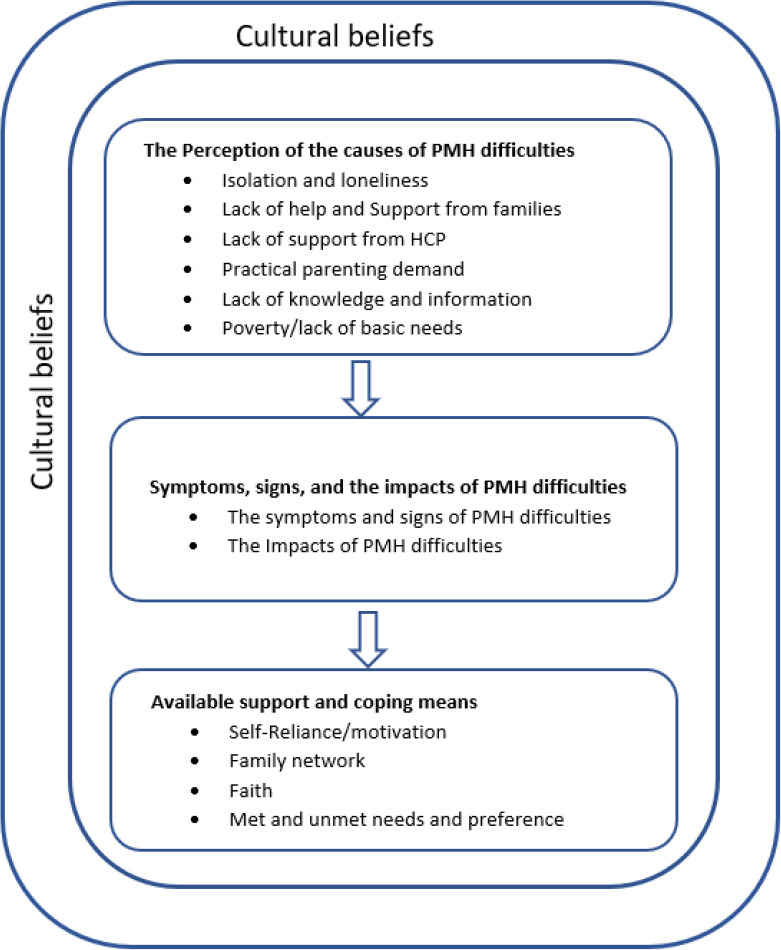
Summary of the themes and subthemes.

The analysis generated three themes (Fig 2): i) perceived causes of PMH difficulties, ii) symptoms, signs and impact of PMH difficulties and iii) available support and coping means. The cultural beliefs of Black immigrant mothers permeate across the three themes.

#### Theme 1: Perceived causes of PMH difficulties.

The focus of this theme was on Black immigrant mothers’ perceptions of the causes of perinatal mental health difficulties. The mothers identified several interrelated factors contributing to their distress such as isolation and loneliness, lack of support from family and healthcare professionals, practical demands associated with parenting, lack of adequate information and poverty/unmet need.

**Isolation and loneliness:** Black immigrant mothers expressed significant concern over feeling isolated and lonely in the UK [38–40].

“Well I have nobody, it’s just like you are an island on your own. I have got nobody to help me” (Participant 5) [38].

Adjusting to the new culture has been described as a feeling of isolation and loss of the comfortable life they were previously used to. The mothers made a comparison between their collectivist culture and the UK being individualistic [39].

“I guess we come from a very communal society where you always have help, always surrounded by someone, and then you come to a society where it’s very individualistic, everybody keeps to themselves. You can’t knock on the next-door neighbour to say, please look after my child, so it does involve culture shock” (Julie) [39].

Additionally, the cultural stigma associated with perinatal mental health (PMH) difficulties and the fear of being labelled as weak or stigmatized made some of these Black immigrant mothers distance themselves from their wider communities [38,39].

“I didn’t just……. open up totally…… to them. I wouldn’t want to…. You know it’s like an African community and I felt you know…. If one person knows about it, 2 people know about….. 3 people knows about it….. so I just cut off. um….. I know it’s just the stigma…. It’s just you know oh… look at the girl….. I think it’s just, it’s just that I don’t want the stigma to just keep following me around” (Participant 5) [38].

**Lack of help and support from family:** Black immigrant mothers expressed that in their home country, it was usual for them to have adequate help and rest after giving birth to their babies. However, they noted that their experiences in the UK differed significantly from their experiences in their country due to a lack of support and the distance from their relatives [37,38,40].

“You are attached to the family house you are not on your own, they make meals for you… in fact you actually don’t do anything. They bathe the baby for you, they help you out with everything until you are fine. And you are ready to go back to work or whatever it is you are doing so… the pressure is very low in Africa, it is not like that here” (Participant 4) [38].

For some of these mothers, their families and husbands were available locally but did not help or support them [38–40].

“I was not very happy with my husband … I felt like he wasn’t giving me the attention that I needed or helping me as much as I needed to because he was always away working, … I hardly see him” (Ngozi) [39].

**Lack of support from health care professionals (HCP):** Lack of support from HCP has been noted to be at several levels, e.g., poor access/counselling, poor quality of services at home/hospital, and child health priority over maternal mental health [37–40].

Some mothers suggested they would have spoken to an external source, such as HCP, if given the opportunity.

“I could have said something if someone had pushed me or asked about how I was coping, I would have been open to talk about it because I think they would have helped me” (Julie) [39].

There was a widespread belief among Black immigrant mothers in the included studies that HCPs do not provide enough support when required. Mothers in three of the studies shared that they had not received a response when they contacted HCPs and viewed that HCPs sometimes gave unhelpful support without adequate information or follow-up [37,39,40].

Black immigrant mothers shared their experiences and perceptions of the healthcare services and described some of the healthcare workers as not being sensitive to their mental health needs, showing neglect and discrimination [38,39].

“I couldn’t speak to my health visitor … I felt she was just going through a checklist … she was using a set of questions; I think she was asking me the wrong questions … I felt she was not empathetic” (Ade) [39].

Some mothers perceived that healthcare providers prioritised the newborn’s care over the mother’s mental health and well-being, leaving the latter with little or no attention [39].

“They would come and look at the child, how I’m looking after her, they were always enquiring about the child, and not my mental health; so, it’s always like your physical health, is your wound healing? Nobody asked about my mental health” (Julie) [39].

**Practical parenting demands:** The practical parenting demands caused a lack of rest and sleep for Black immigrant mothers in two of the studies [37,40]. Some mothers perceived parenting as a challenging experience, particularly in relation to breastfeeding, with some reporting experiencing pain and being very stressed, making it a daunting task [37].

The women reported that whilst they perceived breastfeeding as difficult, their love for their children and the resultant positive effect on the child’s health (from breastfeeding) motivated them to persevere [37].

“Yeah! Breastfeeding is very painful…… Sometimes there would be sore all over the nipples of my breast but the passion and love I have for my baby force me to still feed her” (Fieldwork 2014) [37].

These demands of practical parenting during the perinatal period are considered relevant to maternal mental health difficulties because the confluence of physical exhaustion, sleep deprivation, and continuous caregiving responsibilities increases mental health vulnerability [37].

**Lack of adequate knowledge and information:** Black immigrant mothers showed a lack of adequate information on perinatal mental health difficulties, the choice of treatment and where to seek help [39,40].

Some of the mothers held the belief that PND symptoms do not manifest in their home country and experienced them for the first time after relocating to the UK [38].

“I don’t experience anybody having it [PND in Nigeria]. Cause I don’t have it. I don’t have it in Nigeria. I did not have it in Nigeria……I never knew it exist… you understand? Until I come to this place [UK]” (Participant 1) [38].

The immigrant mothers expressed their concern about not getting information that helps them cope with the challenges of birthing a baby, most especially from the HCP who cannot recognise when they are in pain or distress [40]. Also, some of the mothers stopped or refused medications due to cultural perceptions, lack of adequate information on the treatment and absence of choice of treatment [39].

“Because I didn’t want to see myself as a ‘junkie’ someone that is relying on tablets, I took the medication for a while and … decided not to take it anymore …..be very very honest with you, I was so surprised that he just immediately prescribed antidepressants for me … without explaining things or checking if I would take it” (Ngozi) [39].

**Poverty/unmet basic needs:** In multiple studies, Black immigrant mothers experienced PMH difficulties due to not being able to fulfil basic needs. Financial constraints and accommodation issues were identified in two studies as factors that contributed to these difficulties and challenges [37,38].

“Yeah I know help is at hand…….. but look at me! This house - I don’t have landline. I have a phone. I have no credit on that phone. Even if I am in trouble, who am I going to call” (Participant 1) [38].

In summary, the experiences of Black immigrant mothers in the UK highlight the various challenges they face, which can lead to perinatal mental health (PMH) issues. Many of these mothers feel isolated and often believe they do not receive the necessary support from their families or the healthcare system. This feeling of loneliness is exacerbated by the demands of parenting and a lack of clear information about mental health resources that cater to their needs.

#### Theme 2: Symptoms, signs and impacts of PMH difficulties.

Black immigrant mothers described various ways in which they experience PMH difficulties and the effects that these difficulties have on their lives.

**Symptoms and signs of PMH difficulties:** The Black immigrant mothers described the symptoms and signs as including excessive crying, sadness, annoyance with self, suicidal thoughts and feelings of hopelessness [37–40].

“I thought to myself I want to jump outside the window with the baby um, then I thought …,it was just a feeling of hopelessness yeah, especially thinking about and wanting to kill yourself but fortunately for me I just thought about the impact on my family and who will look after my children” (Rosemary) [39].

According to Gardner et al. [38] some mothers exhibited symptoms such as excessive self-criticism, low self-esteem, self-harming thoughts, and difficulty sleeping.

“I snapped all the time, unnecessary things would just get on me and I would just fly up (severe depression) dying…… taking my life…. ending it up. I was…. The time it was the peak of the stress I felt like just disappearing into thin air” (Participant 5) [38]

Several women did not recognize their symptoms as indicators of postnatal depression (PND) but instead considered them as everyday stress [38].

“You just see yourself doing things……….. just see yourself talking to yourself, having different thoughts, hearing voices…. … I mean those are all symptoms that I was stressed” (participant 5) [38].

**Impacts of PMH difficulties:** The Black immigrant mothers described the impact perinatal mental health difficulties have on them, and as part of the impact, some of the mothers discussed their inability to cope with the difficulties [37–40].

The Black mothers felt alone with their feelings, believing that no one would understand or help them with the feelings.

“It’s something nobody else can help you with. Like someone can help you with the baby and help you with other things, but the way that you’re feeling, you don’t get help for that.” “That I’m going mad, mentally and sometimes I’d be crying... the baby and I will be crying, and sometimes I feel like throwing the baby out, but I can’t” [40].

In one study, the mothers reported that the pregnancy process negatively impacted their self-esteem, largely due to excessive weight gain and the demands of parenting. They also felt that pregnancy led them to lose their sense of self, often comparing who they were before pregnancy to who they had become after giving birth [38].

“Oh God….. I hate myself. I really, really hate myself. I put on weight, because… and I am always very very conscious of my body. Always you looking at yourself in the mirror and you just don’t like what you see” (Participant 1) [38].

According to one of the Ghanaian immigrant mothers, the demands of taking care of the newborn led to emotional distress and exhaustion and the compromise of self-care and that of the other child [37].

“I sometimes get fed up with the constant and persistent cries of my baby. He always needs attention and I can’t do anything for myself and my 4-year-old son. It gets me angry at times and I leave him to cry. However, I feel sad when the baby cries especially when I have to take my 4-year-old son to school”(Fieldwork 2014) [37].

Some mothers raised concerns about difficulty coping [39,40].

“so the first week was very difficult for me to cope with changing him, feeding him, it wasn’t easy, it was my first time, so I would cry sometimes; and not until when my mum came, things were a little bit easier for me. But I couldn’t really cope with the emotions. Sometimes like I said, it was mixed feelings” [40].

Also, because of the cultural taboo associated with depression and other mental health issues, some Nigerian mothers shared that, whilst they were not able to cope, they pretended to be fine [39].

Black immigrant mothers face significant challenges related to perinatal mental health (PMH) issues, which often go unrecognised and untreated. Many reported symptoms, such as excessive crying, hopelessness, and suicidal thoughts, may be viewed by mothers as related to everyday stress rather than signs of postnatal depression. Their experiences often lead to feelings of isolation and low self-esteem, particularly as they juggle the demands of motherhood and lose their sense of identity after childbirth. Further compounding are cultural taboos surrounding mental health, which may prevent mothers from seeking the help they need. There is a clear need for better support systems that understand and address the unique challenges these mothers face, helping them manage their mental health effectively.

#### Theme 3: Available support and coping means.

Following an exploration of the causes, manifestations, and impacts of mental health challenges faced during the perinatal period, some Black immigrant mothers shared the various support systems available to them and the strategies they employed to navigate these difficulties including their met or unmet needs.

**Self-reliance/ motivation:** Most Black immigrant mothers viewed depression and stress as an inherent part of motherhood, and as a result, they often relied on positive thinking and self-help strategies rather than seeking professional help [37–39].

“For me when I am down, I just want to do something, go out for window shopping or sometimes tidy the house. But the best thing for me is to go out………. What I did, I went out and I got a salary - a shop, I start working and all of a sudden it took my mind off it” (Participant 2) [38].

One study reported that mothers motivated themselves by thinking of the impact on the family and the children [39].

“I just felt I can’t feel like this because I’ve got [you know], the children, so I needed to take care of them, and I needed to take care of myself” (Rosemary) [39].

Also, as a coping mechanism, West African mothers commented that bonding with and delighting in their babies helped to distract them from painful feelings and experiences [38].

“There are so many things that she does (the baby) that just make me you know, forget my sorrows, forget the pains I am going through” (Participant 2) [38].

**Family network:** The Black immigrant mothers reported that some of their families, who were available in the UK, were able to offer support similar to that which they would have received in their home country [37,38]. This support, coupled with seeking out other mothers in their communities for information, reassurance, and support, helped them cope well with their difficulties [38].

“Any little problem I had I used to call my friends,…… [I would] say this is what has happened….. then they say ‘ok it’s a normal thing’, it happens to [them] - just advice from other parent” (Participant 4) [38].

Some mothers preferred to seek advice from their family members in their home country by phone, as they felt that reaching out to an HCP would not be of much help.

“…………So, I talked to my mum back home, I have to call my mum. I spend a lot of money buying phone cards to talk to my mum. Every day, I am on the phone with my mum 24/7, every sec, every minute, even if my baby is crying too much I have to call my mum and ask for advice” (Fieldwork 2014) [37]

**Faith:** Some Black immigrant mothers in one of the studies expressed a high level of confidence in their ability to cope with struggles and difficulties, which they attribute to their belief in God. They reported that their faith had been a crucial motivating factor that has sustained them through challenging times [38].

“…. it was my belief and faith in God, cause I kept praying, my church prayed for me at all times and all that…… and I believed that I would be well again, it was in my head…..so it was………. my faith in God” (Participant 6) [38].

**Met and unmet needs and preferences:** Black immigrant mothers reflected on the support and help available to new mothers in their communities, where family members come to stay with the new mothers to provide support and help in the best way possible until the new mothers had fully readjusted and recovered. The absence of such support or something relatively similar in the UK had left them feeling let down. They suggested that having a community of mothers with whom they could connect would provide similar support to what they would have received in Africa and help them manage any difficulties that come with pregnancy [38].

“……… [when you start going to the group] you know that you are not alone. So many mothers are going through what you are going through. And some are even MORE than yourself…….. [I think] there should be a gathering for mothers……. So you can chat with another mother.…. it does help” (Participant 1) [38].

The mothers shared their experience of adjusting to unfulfilled expectations and coping with the disappointment from HCPs. Despite feeling helpless and defeated, the mothers allowed the professionals to carry out their duties and personally managed the difficulties as best as they could without professionals’ support [39].

“……….and when you know they are coming you just put a smiling face, show them the things they want to see … They come and look at the environment, they look at the baby, and off they go … Then when they go, you now have to go back to where you started from” (Blessing) [39].

This theme showcases the systemic and sociocultural supports available to this demographic, the coping mechanisms employed and the various met and unmet needs.

## Discussion

This review illustrates the experiences of Black immigrant mothers in the UK regarding perinatal mental health (PMH) difficulties. It identifies three key themes: perceived causes of PMH issues, their symptoms and impacts, and available support and coping strategies, with cultural beliefs playing a significant role. Many mothers experience feelings of isolation and loneliness due to a lack of family and healthcare support, compounded by practical parenting demands and cultural believe surrounding mental health. Symptoms such as excessive crying, suicidal thought and hopelessness often go unrecognized, which affect their mental well-being and identity. The review shows the need for culturally competent support systems to address their unique challenges and improve mental health resources for these mothers.

The included studies indicated that Black immigrant mothers’ cultural perceptions and beliefs of being resilient, self-sufficient and courageous under challenging situations may hinder them from seeking help and discussing their mental health. This is to avoid being labelled as a weak mother or a disappointment to other family members. Studies have shown that mental health stigma is more common in Black women than white women [41], with an inverse relationship between stigma and help-seeking behaviour [20,42]. Also, a study by Watson and Soltani [9] with women from minority ethnic groups in England reported that the stigma and cultural norms surrounding postpartum mental health issues made it difficult for them to discuss their PMH problems openly. Collectively, the literature suggests that a significant proportion of women from minority ethnic groups tend to remain silent about their mental health issues, and those who do attempt to seek help often encounter inadequate support [9]. This review suggests consistent findings across research with minority ethnic women and Black immigrant women.

The perceived causes of PMH difficulties are consistent with other studies that involves mothers from minoritised ethnic group and other Black mothers [43–45]. Although some Black immigrant mothers can identify the causes and symptoms of perinatal mental health difficulties, lack of adequate information among them, their families and the community on perinatal mental difficulties often leads to inappropriate action or no action at all. It has been acknowledged that high-quality and culturally sensitive information must be provided to every woman to help differentiate between typical symptoms of pregnancy and perinatal mental health symptoms [46]. Both perceived stigma and mental health literacy have been proven to be associated with responses towards help-seeking from healthcare services [47]. Therefore, it is essential to educate women, families, and the community about PMH in a culturally competent way. This can help raise awareness and support and encourage family members to offer more assistance.

In this review, the experiences of support from healthcare professionals, family and community have not been encouraging, as some mothers described being neglected and not getting a response from the healthcare services when in need. This finding is congruent with a report, “Systemic Racism, Not Broken Bodies,” describing racial injustice and human rights in the UK Maternity Service [48]. Healthcare professionals’ lack of confidence and training needs have also been identified in recognising Black mothers’ perinatal mental health needs and managing them appropriately [28]. Whilst some women from minoritised ethnic groups in the UK perceived healthcare professionals and community-based professionals as potential sources of help [9], inequalities in accessing perinatal mental health (PMH) care continued; shaped by aspects such as the presence of stigma, the lack of trust in healthcare services, as well as the attitudes of healthcare providers towards the women [49].

The review highlights the coping strategies adopted by Black immigrant mothers to prevent or reduce the effect of distress are consistent with older literature with Black women who were UK-born but described as settled immigrants [50,51] and the study of Amankwaa [52]with African American mothers, where some participants mentioned faith, prayer and help from families as major coping strategies. These studies confirmed that family support and religious resources can be beneficial to meeting the needs of Black women [50–52]. This points to the importance of further consideration of intersectionality in research and practice, including the impact of religion and other factors on maternal mental health, especially for new immigrant mothers who lack immediate family support and discontinued social connections in the host country. Some charities and community initiatives in the UK are now available in the voluntary sector, intended to provide support to Black mothers in recognition of the considerable health disparities. It is possible that these may meet the needs of women who are unable to access health services however there can be local variation in provision and formal evaluation is warranted.

A growing body of literature has established that both immigration and racism have significant negative impacts on mental health. For immigrants, mental distress is often associated with acculturative stress, social isolation, loss of status, and uncertainty about legal status or access to services [53,54]. When applied to the perinatal mental health experiences of Black immigrant mothers, these women often face challenges such as acculturative stress, social isolation, and worries about their legal status, which are compounded by linguistic barriers and a lack of culturally competent healthcare services. Stigma surrounding mental illness in many African and Caribbean communities further complicates their ability to seek support [8] however this has also been founded with other minoritised groups. The intersectionality of race and immigration status creates unique vulnerabilities, resulting in restricted access to care and diminished trust in healthcare providers [15].

Some studies were not eligible for inclusion in this review as it included Black women born in the UK, who would be expected to have a good level of knowledge and awareness about the health system and a more established support system as the settled residents of the country they are born. Nonetheless, the findings from this review aligned with those of this review, most notably Edge’s work with Black Caribbean women [8,51,55] which raised concerns about insensitive perception of meeting their needs and health professionals’ lack of compassion during pregnancy which resulted in doubt of seeking help. This raises the question of whether Black women in the UK have comparable experiences of PMH difficulties regardless of whether they are British-born or immigrated Black women in the UK. Although this review could not answer this particular question, it shows the necessity for future empirical studies to specifically target Black immigrant mothers in the UK, so that the comparison could be made.

### Implications for research

Despite an inclusive approach to perinatal mental health in the searching and selection strategies, included studies were limited to postnatal depression, albeit with some occasional mentions of antenatal depression and other perinatal mental health difficulties. There is an urgent need for further empirical research to examine perinatal mental health more widely, including anxiety and trauma, with Black immigrant mothers. Research is lacking on the support and services from family and the community for Black immigrant mothers during the perinatal period and how their relationship with their child affects and is affected by their mental health. There is a need to focus on the family and community regarding perceptions of supporting mothers with their perinatal mental health.

An area requiring further exploration is the role of bonding in Black immigrant mothers as a protective factor against PMH, indicated by one study [38]. This finding is consistent with previous research with Black Caribbean women in the UK [51], who viewed their relationships with their children as a protective mechanism against depression or as a source of motivation for managing the condition. However, this is likely a complex area and the review also includes comments relating to the challenges of practical parenting demands and availability to children because of the competing priorities, highlighting varied experiences. Given the potential implications of maternal mental health difficulties for children [56,57], further research is needed on the parents’ perspectives on the interactions between parent mental health, parent-infant relationship and children’s development.

Clinically, the research reflects the need for more training in culturally sensitive and antiracist care practices, adapting perinatal mental health assessment to reflect different cultural contexts and experiences. Also, integrating a peer-led and community-based models into the care pathways is vital as these models provide trusted support and help connect marginalised communities with the healthcare system.

### Strengths and limitations

The review focuses on the perinatal mental health experiences of Black immigrant mothers in the UK, which has a significant population of Black immigrants from Africa and the Caribbean. This approach aims to address the unique cultural and health challenges these mothers face within the UK’s complex context, despite the country’s universal healthcare system. Notably, Black women in the UK encounter significant health disparities.

The review process involved multiple reviewers to ensure a comprehensive and systematic approach. Although the themes aligned closely with the research question, this was considered appropriate. [35] acknowledged that thematic synthesis can involve synthesis without new conceptual innovation if the primary studies address the review question directly. Only a limited number of studies featuring Black immigrant mothers were found and these cannot be interpreted as representative of the wider population, particularly with all having been limited to Black African mothers; moreover, the majority of participants were from West Africa, specifically Ghana and Nigeria. No studies with Black Caribbean women were eligible due to none being focused on women born outside the UK. It was, therefore, not possible to consider further heterogeneity within Black immigrant mothers in the UK. Nonetheless, the high methodological quality of the studies included enhances the confidence and trustworthiness of the results obtained.

## Conclusion

The review, intended to understand the perinatal mental health experiences of Black immigrant mothers in the UK, firstly shows the lack of available evidence. Furthermore, the available evidence is focused on postnatal depression, despite growing understanding of the importance of a wider approach to perinatal mental health. The findings suggest a lack of non-readily available support, lack of adequate information, and lack of support from family, community and healthcare professionals, and a range of individual approaches to coping; all shaped by cultural perceptions. Synthesising the voices of Black immigrant mothers across studies demonstrates the need for perinatal mental health support services to fully understand the cultural aspects to meet mothers’ needs and provide accessible and acceptable services. However, these findings are consistent with wider literature with minoritised ethnic communities and with migrants and do not offer any distinct considerations for this specific population. The needs of Black African immigrant mothers and Black Caribbean immigrant mothers may be comparable; however, exploration is precluded due to a lack of available literature, limited to mothers from West Africa, specifically Nigeria and Ghana. Research with Black immigrant mothers urgently needs to widen beyond postnatal depression and include greater consideration of parent-infant relationships and wider support needs to inform culturally competent services.

## Supporting information

S1 Table Search strategy. (DOCX)

S2 Table CERQual summary of qualitative findings. (DOCX)

S3 Table CERQual qualitative evidence profile. (DOCX)

S1 Checklist Prisma checklist. (DOCX)
