# Supplementary material for: The perinatal mental health experiences of black immigrant mothers in the UK: A qualitative systematic review and thematic synthesis
Source: PLoS One. 2025 Dec 19;20(12):e0331547. doi: 10.1371/journal.pone.0331547 (PMC12716732; doi:10.1371/journal.pone.0331547)
Supplement: S1 Table — (DOCX) [file pone.0331547.s001.docx]

Supporting Information 1: Search Terms and Combination.

| 1 | Perinatal | 9 | Anxiety | 17 | Black | 25 | UK | 33 | 14 or 15 or 16 |
| --- | --- | --- | --- | --- | --- | --- | --- | --- | --- |
| 2 | Postnatal | 10 | Stress | 18 | African | 26 | United Kingdom | 34 | 17 or 18 or 19 or 20 |
| 3 | Antenatal | 11 | Wellbeing | 19 | Caribbean | 27 | England | 35 | 21 or 22 or 23 or 24 |
| 4 | Postpartum | 12 | Emotion* | 20 | Afro-Caribbean | 28 | Wales | 36 | 25 or 26 or 27 or 28 or 29 or 30 |
| 5 | Maternal | 13 | Psychology | 21 | Immigrant | 29 | Scotland | 37 | 31 and 32 and 33 and 34 and 35 |
| 6 | Pregnant* | 14 | Experiences | 22 | Migrant | 30 | Northern Ireland | 38 | 37 limited to English |
| 7 | Mental health | 15 | View | 23 | Refugee | 31 | 1 or 2 or 3 or 4 or 5 or 6 | 39 | 37 limited to English in Medline = 135 articles |
| 8 | Depression | 16 | Perception | 24 | Asylum | 32 | 7 or 8 or 9 or 10 or 11 or12 or 13 | 40 | 37 limited to English in CINAHL=24 |

| Database | Search Terms | Search Limit | Number of Articles |
| --- | --- | --- | --- |
| PscyINFO | (Perinatal OR Postnatal OR Antenatal OR Postpartum OR Maternal OR Pregnan*) AND (Mental health OR Depression OR Anxiety OR stress OR Wellbeing OR Emotion OR Psychology) AND (Experiences OR View* OR Perception) AND (Black OR African OR Caribbean OR Afro-Caribbean) AND (Migrants OR Immigrants OR Refugees OR Asylum) | Limited to English. | 66 |
| MAG online Library | Perinatal OR Postnatal OR Antenatal OR Postpartum OR Maternal OR Pregnan***AND**Mental health OR Depression OR Anxiety OR distress OR stress OR Wellbeing OR Emotion OR Psychology**AND**Experiences OR View OR Percept***AND**Black OR African OR Caribbean OR Afro-Caribbean**AND**Migrants OR Immigrants OR Refugees OR Asylum | Limited to English | 167 |
| Scopus | (perinatal OR postnatal OR antenatal OR postpartum OR Maternal OR pregnan*) AND (TITLE-ABS-  KEY (mental AND health OR depression OR anxiety OR stress OR wellbeing OR emotion OR psychology) ) AND (TITLR-ABS-  KEY (experiences OR view* OR perception) ) AND (TITLE-ABS  KEY (black OR African OR Caribbean OR Afro-caribbean) ) AND (TITLE-ABS-KEY (migrants OR immigrants OR refugees OR asylum) ) | Limited to English | 22 |
