# Supplementary material for: The perinatal mental health experiences of black immigrant mothers in the UK: A qualitative systematic review and thematic synthesis
Source: PLoS One. 2025 Dec 19;20(12):e0331547. doi: 10.1371/journal.pone.0331547 (PMC12716732; doi:10.1371/journal.pone.0331547)
Supplement: S2 Table — (DOCX) [file pone.0331547.s002.docx]

**Supporting Information 2**

**S2**. CERQual Summary of Qualitative Findings

| **Review Findings** | **Studies Contributing to the Review Findings** | **Example for data support (original citations from the qualitative studies’ participants)** | **CERQual Assessment of Confidence in the Evidence (the likelihood that the finding is a reasonable representation of the phenomenon of interest)** | **Explanation of CERQual Assessment** |
| --- | --- | --- | --- | --- |
| **The Perception of the causes of PMH difficulties** | | | | |
| **Isolation and loneliness**  Black immigrant mothers in the UK often feel isolated and lonely while adjusting to a new culture. They compare the collectivist culture they came from to the individualistic culture of the UK. They also fear being stigmatized for perinatal mental health difficulties, causing them to distance themselves from their communities. | [38-40] | “Well I have nobody, it's just like you are an island on your own. I have got nobody to help me” [38]. | **High** | Three studies contributed to this review finding. There were no or very minor concerns regarding methodological limitations, relevance coherence and adequacy. |
| **Lack of help and support from families**  Some Black immigrant mothers in the UK feel that they lack adequate help and support after giving birth, which is a significant difference from their experiences in their home countries. Some have family and husbands nearby but do not receive help or support from them. | [37-40] | “I was not very happy with my husband … I felt like he wasn’t giving me the attention that I needed or helping me as much as I needed to because he was always away working, … I hardly see him” [39]. | **High** | Four studies contributed to this review finding. There were no or very minor concerns regarding methodological limitations, relevance coherence and adequacy. |
| **Lack of support from HCP**  Black immigrant mothers report a lack of support from healthcare providers at different levels, such as poor access to counselling and poor quality of services. Some HCPs are perceived as prioritizing child health over maternal mental health, and some mothers feel that they do not get adequate support or follow-up. Additionally, some healthcare workers are perceived as being insensitive and discriminatory towards the mental health needs of new mothers. | [37-40] | “I couldn’t speak to my health visitor … I felt she was just going through a checklist … she was using a set of questions; I think she was asking me the wrong questions … I felt she was not empathetic” [39]. | **High** | Four studies contributed to this review finding. There were no or very minor concerns regarding methodological limitations, relevance coherence and adequacy. |
| **Practical parenting demand**  Black immigrant mothers struggle with a lack of rest due to parenting demands. Some Black immigrant mothers find breastfeeding particularly challenging, causing pain and discomfort. However, their love for their children and the positive impact on their health motivates them to persevere. | [37,40] | “Yeah! Breastfeeding is very painful…… Sometimes there would be sore all over the nipples of my breast but the passion and love I have for my baby force me to still feed her” [37]. | **Moderate** | Two studies contributed to the finding. There are no concerns about methodological limitations, relevance, or coherence but moderate concerns about adequacy. |
| **Lack of knowledge and information**  Immigrant mothers lack information on perinatal mental health, treatment, and where to seek help. Some believe PND symptoms don't manifest in their home country and face them for the first time in the UK. There is a concern about not getting adequate information to cope with birthing challenges, and some mothers refuse medication due to cultural perceptions and lack of information on treatment. | [38-40] | “Because I didn’t want to see myself as a ‘junkie’ someone that is relying on tablets, I took the medication for a while and … decided not to take it anymore …..be very very honest with you, I was so surprised that he just immediately prescribed antidepressants for me … without explaining things or checking if I would take it” [38]. | **High** | Three studies contributed to this review finding. There were no or very minor concerns regarding methodological limitations, relevance coherence and adequacy. |
| **Poverty/lack of basic needs**  Black immigrant mothers experienced difficulties with their mental health due to unmet basic needs. Financial constraints and housing issues were identified as other factors contributing to these challenges. | [37-38] | “Yeah I know help is at hand…….. but look at me! This house - I don't have landline. I have a phone. I have no credit on that phone. Even if I am in trouble, who am I going to call” [38] | **Moderate** | Two studies contributed to the finding. There are no concerns for the methodological limitation, relevance and coherence but moderate concern for the adequacy. |
| **The Symptoms, Signs and Impacts of PMH Difficulties** | | | | |
| **The symptoms and signs of PMH difficulties**  The Black immigrant mothers described the symptoms and signs as excessive crying, sadness, annoyance with self, suicidal thoughts and feelings of hopelessness. Some mothers exhibited symptoms such as excessive self-criticism, low self-esteem, self-harming thoughts, and difficulty sleeping. | [37-40] | “You just see yourself doing things……….. just see yourself talking to yourself, having different thoughts, hearing voices…. … I mean those are all symptoms that I was stressed” [38]. | **High** | Four studies contributed to this review finding. There were no or minor concerns regarding methodological limitations, relevance coherence and adequacy. |
| **The impacts of PMH difficulties**  Black immigrant mothers reported negative impacts on their self-esteem, sense of self, and ability to cope. Some mothers have also experienced neglect and difficulty caring for their other children. Additionally, cultural taboos surrounding mental health have caused some of these mothers to pretend to be fine even when they are struggling. | [37-40] | “I sometimes get fed up with the constant and persistent cries of my baby. He always needs attention and I can’t do anything for myself and my 4-year-old son. It gets me angry at times and I leave him to cry. However, I feel sad when the baby cries especially when I have to take my 4-year-old son to school” [37]. | **High** | Four studies contributed to this review finding. There were no or minor concerns regarding methodological limitations, relevance coherence and adequacy. |
| **Available Support and coping means** | | | | |
| **Self-reliance/motivation**  Black immigrant mothers often view depression and stress as an inherent part of motherhood and rely on positive thinking and self-help strategies. Some mothers motivate themselves by considering the impact on their family and children. Bonding with their babies helps distract them from painful feelings and experiences. | [37-39] | “For me when I am down, I just want to do something, go out for window shopping or sometimes tidy the house. But the best thing for me is to go out………. What I did, I went out and I got a salary - a shop, I start working and all of a sudden it took my mind off it” [38]. | **High** | Three studies contributed to this review finding. There were no or minor concerns regarding methodological limitations, relevance coherence and adequacy |
| **Family Network**  Some Black immigrant mothers have found support from their families in the UK and their communities to help them cope with their difficulties. Some prefer to seek advice from family members back home instead of healthcare professionals. | [37,38] | “…………So, I talked to my mum back home, I have to call my mum. I spend a lot of money buying phone cards to talk to my mum. Every day, I am on the phone with my mum 24/7, every sec, every minute, even if my baby is crying too much I have to call my mum and ask for advice” [37]. | **Moderate** | Two studies contributed to the finding. There are no concerns for the methodological limitation, relevance and coherence but moderate concern for the adequacy. |
| **Faith**  Some Black immigrant mothers have reported that their belief in God has given them the confidence and motivation to cope with struggles and difficulties. | [38] | “…. it was my belief and faith in God, cause I kept praying, my church prayed for me at all times and all that…… and I believed that I would be well again, it was in my head…..so it was………. my faith in God” [38]. | **Moderate** | Only a study contributed to the finding. There are no concerns for the methodological limitation, relevance and coherence but moderate concern for the adequacy. |
| **Met and unmet needs and preferences**  Black immigrant mothers miss the support they receive from family members in their African communities during the postpartum period. They suggest having a community of mothers to connect with in the UK would be helpful. Some have struggled with unfulfilled expectations and disappointment from healthcare professionals but managed the difficulties as best they could. | [38, 39] | “……… [when you start going to the group] you know that you are not alone. So many mothers are going through what you are going through. And some are even MORE than yourself…….. [I think] there should be a gathering for mothers……. So you can chat with another mother.…. it does help” [38]. | **Moderate** | Two studies contributed to the finding. There are no concerns for the methodological limitation, relevance and coherence but moderate concern for the adequacy. |

37. Dei-Anane E, Poku AA, Boateng S, Poku KO, Amankwa E, Adasa AN, et al. Perceptions of Ghanaian migrant mothers living in London towards postnatal depression during postnatal periods. Am J Geogr Res Rev. 2018;2:1-8. doi: 10.28933/ajgrr-2018-02-0501.

38. Gardner PL, Bunton P, Edge D, Wittkowski A. The experience of postnatal depression in West African mothers living in the United Kingdom: a qualitative study. Midwifery. 2014;30(6):756-63. doi: 10.1016/j.midw.2013.08.001. PubMed PMID: 24016554.

39. Ling L, Eraso Y, Mascio VD. First-generation Nigerian mothers living in the UK and their experience of postnatal depression: an interpretative phenomenological analysis. Ethn Health. 2022;.doi: 10.1080/13557858.2022.

40. Babatunde T, Moreno-Leguizamon CJ. Daily and cultural issues of postnatal depression in African women immigrants in South East London: tips for health professionals. Nur Res Pract. 2012;2012:181640. doi: 10.1155/2012/181640. PubMed PMID: 23056936.
