# Supplementary material for: The perinatal mental health experiences of black immigrant mothers in the UK: A qualitative systematic review and thematic synthesis
Source: PLoS One. 2025 Dec 19;20(12):e0331547. doi: 10.1371/journal.pone.0331547 (PMC12716732; doi:10.1371/journal.pone.0331547)
Supplement: S3 Table — (DOCX) [file pone.0331547.s003.docx]

**Supporting Information 3**

S3. CERQual Qualitative Evidence Profile

| **Findings** | **Contributing study** | **Assessment for each CERQual component** | | | | **Confidence**  **in the**  **evidence** | **Overall CERQual assessment** |
| --- | --- | --- | --- | --- | --- | --- | --- |
|  |  | **Methodological Limitation** | **Relevance** | **Coherence** | **Adequacy** |  |  |
| **The Perception of the causes of PMH difficulties** | | | | | | | |
| **Isolation and loneliness** | [38-40] | **No or very minor concerns**  There is a concern regarding the reflexivity of Gardner et al. [38] (the relationship between the researcher and the participants). However, the credibility of the findings was not impacted by the lack of information about the researcher's role and relationship with the participants. Hence, the review's supporting evidence was evaluated as having minimal or no concerns regarding methodological limitations. | **No or very minor concern**.  Although two of the studies did not primarily focus on the perinatal mental health experience of mothers, they both shared the narratives of the mothers, which proved to be a significant contribution to the review's overall findings. One of the studies explored the perception of Postnatal depression (PND), while the other delved into the daily and cultural challenges faced by mothers with PND. Despite their different focus, both studies provided valuable insights into the perinatal mental health experiences of mothers. | **No concerns**  Three out of four studies have contributed to this review, and there is a clear and coherent alignment of the study with the review's finding. The studies accurately reflect the phenomenon of interest of the review, and there are no variations or exceptions in the data. | **No concerns**  Three studies contributed to this review finding, providing a variety of examples that illustrate and explain the finding | **High** | Three studies contributed to this review finding. There were no or very minor concerns regarding methodological limitations, relevance coherence and adequacy. |
| **Lack of help and support from families** | [37-40] | **No or very minor concerns**  There is a concern regarding the reflexivity of Dei-Anane et al. [37] and Gardner et al. [38] (the relationship between the researcher and the participants). However, the credibility of the findings was not impacted by the lack of information about the researcher's role and relationship with the participants. Hence, the review's supporting evidence was evaluated as having minimal or no concerns regarding methodological limitations. | **No or very minor concerns**  Although two of the studies did not primarily focus on the perinatal mental health experience of mothers, they both shared the narratives of the mothers, which proved to be a significant contribution to the review's overall findings. One of the studies explored the perception of Postnatal depression (PND), while the other delved into the daily and cultural challenges faced by mothers with PND. Despite their different focus, both studies provided valuable insights into the perinatal mental health experiences of mothers. | **No or minor concerns**  All four studies contributed to this review and provided various examples supporting the findings. Although some findings did not directly describe the review findings, they provided rich underlying data. | **No concerns**  All four studies provided detailed and specific information regarding the findings. Based on the overall assessment of data richness and quantity, we have no concerns about data adequacy. | **High** | Four studies contributed to this review finding. There were no or very minor concerns regarding methodological limitations, relevance coherence and adequacy. |
| **Lack of support from healthcare professionals** | [37-40] | **No or very minor concerns**  There is a concern regarding the reflexivity of Dei-Anane et al. [37] and Gardner et al. [38] (the relationship between the researcher and the participants). However, the credibility of the findings was not impacted by the lack of information about the researcher's role and relationship with the participants. Hence, the review's supporting evidence was evaluated as having minimal or no concerns regarding methodological limitations. | **No or very minor concerns**  Although two of the studies did not primarily focus on the perinatal mental health experience of mothers, they both shared the narratives of the mothers, which proved to be a significant contribution to the review's overall findings. One of the studies explored the perception of Postnatal depression (PND), while the other delved into the daily and cultural challenges faced by mothers with PND. Despite their different focus, both studies provided valuable insights into the perinatal mental health experiences of mothers. | **No or minor concerns**  All four studies contributed to this review and provided various examples supporting the findings. Although some findings did not directly describe the review findings, they provided rich underlying data. | **No concerns**  All four studies provided detailed and specific information regarding the findings. Based on the overall assessment of data richness and quantity, we have no concerns about data adequacy. | **High** | Four studies contributed to this review finding. There were no or very minor concerns regarding methodological limitations, relevance coherence and adequacy. |
| **Practical parenting demands** | [37,40] | **No or very minor concerns**  There is a concern regarding the reflexivity of Dei-Anane et al. [37] (the relationship between the researcher and the participants). However, the credibility of the findings was not impacted by the lack of information about the researcher's role and relationship with the participants. Hence, the review's supporting evidence was evaluated as having minimal or no concerns regarding methodological limitations. | **No or minor concerns**  The two studies in this finding did not directly focus on perinatal mental health experiences. One studied postnatal depression perception, and the other delved into daily and cultural issues faced by mothers with PND. However, both included narratives of mothers' experiences that significantly contributed to the findings. | **No or minor concerns**  Although the core finding was only directly supported by one study, the other one indirectly described it or did not explore it in detail. However, there were no contradictory data. | **Moderate concerns**  The findings were based on two studies, but only one provided detailed information. The other study contributed partially to the findings, leading to moderate concern about the adequacy of the data. | **Moderate** | Two studies contributed to the finding. There are no concerns for the methodological limitation, relevance and coherence but moderate concern for the adequacy. |
| **Lack of knowledge and information** | [38-40] | **No or very minor concerns**  There is a concern regarding the reflexivity of Gardner et al. [38] (the relationship between the researcher and the participants). However, the credibility of the findings was not impacted by the lack of information about the researcher's role and relationship with the participants. Hence, the review's supporting evidence was evaluated as having minimal or no concerns regarding methodological limitations. | **No or very minor concerns**  One out of the three studies that contributed to this finding did not directly focus on the perinatal mental health experience of the mothers. The focus was on the daily and cultural issues of the mothers on PND. However, it demonstrated the experiences of these mothers during the period, thus contributing significantly to the review's findings. | **No or minor concerns**  Three out of four studies have contributed to this review, and there is a clear and coherent alignment of the study with the review's finding. The studies accurately reflect the phenomenon of interest of the review, and there are no variations or exceptions in the data. | **No or minor concerns**  Three studies contributed to this review finding, providing a variety of examples that illustrate and explain the finding | **High** | Three studies contributed to this review finding. There were no or very minor concerns regarding methodological limitations, relevance coherence and adequacy. |
| **Poverty/lack of basic needs** | [37,38] | **No or very minor concerns**  There is a concern regarding the reflexivity of Dei-Anane et al. [37] and Gardner et al. [38] (the relationship between the researcher and the participants). However, the credibility of the findings was not impacted by the lack of information about the researcher's role and relationship with the participants. Hence, the review's supporting evidence was evaluated as having minimal or no concerns regarding methodological limitations. | **No or minor concerns**  One of the two studies that contributed to this finding did not directly study the perinatal mental health experience of the mothers. Instead, it focused on the perceptions of Ghanaian mothers towards Postnatal Depression (PND). However, it still provided insight into the experiences of these mothers during that period and contributed significantly to the review's findings. | **No concerns**  There is a clear and cogent fit of the two studies into the review findings | **Moderate concerns**  Two studies contributed to this review, providing examples that illustrate and explain the finding. We concluded that we have moderate concerns about the data adequacy. | **Moderate** | Two studies contributed to the finding. There are no concerns for the methodological limitation, relevance and coherence but moderate concern for the adequacy. |
| **The Symptoms, Signs and Impacts of PMH Difficulties** | | | | | | | |
| **The symptoms and signs of PMH difficulties** | [37-40] | **No or very minor concerns**  There is a concern regarding the reflexivity of Dei-Anane et al. [37] and Gardner et al. [38] (the relationship between the researcher and the participants). However, the credibility of the findings was not impacted by the lack of information about the researcher's role and relationship with the participants. Hence, the review's supporting evidence was evaluated as having minimal or no concerns regarding methodological limitations. | **No or very minor concerns**  Although two of the studies did not primarily focus on the perinatal mental health experience of mothers, they both shared the narratives of the mothers, which proved to be a significant contribution to the review's overall findings. One of the studies explored the perception of postnatal depression (PND), while the other delved into the daily and cultural challenges faced by mothers with PND. Despite their different focus, both studies provided valuable insights into the perinatal mental health experiences of mothers. | **No or minor concerns**  All four studies contributed to this review and provided various examples supporting the findings. Although some findings did not directly describe the review findings, they provided rich underlying data. | **No concerns**  All four studies provided detailed and specific information regarding the findings. Based on the overall assessment of data richness and quantity, we have no concerns about data adequacy. | **High** | Four studies contributed to this review finding. There were no or minor concerns regarding methodological limitations, relevance coherence and adequacy. |
| **The impacts of PMH difficulties** | [37-40] | **No or very minor concerns**  There is a concern regarding the reflexivity of Dei-Anane et al. [37] and Gardner et al. [38] (the relationship between the researcher and the participants). However, the credibility of the findings was not impacted by the lack of information about the researcher's role and relationship with the participants. Hence, the review's supporting evidence was evaluated as having minimal or no concerns regarding methodological limitations. | **No or very minor concerns**  Although two of the studies did not primarily focus on the perinatal mental health experience of mothers, they both shared the narratives of the mothers, which proved to be a significant contribution to the review's overall findings. One of the studies explored the perception of Postnatal depression (PND), while the other delved into the daily and cultural challenges faced by mothers with PND. Despite their different focus, both studies provided valuable insights into the perinatal mental health experiences of mothers. | **No or minor concerns**  All four studies contributed to this review and provided various examples supporting the findings. Although some findings did not directly describe the review findings, they provided rich underlying data. | **No concerns**  All four studies provided detailed and specific information regarding the findings. Based on the overall assessment of data richness and quantity, we have no concerns about data adequacy. | **High** | Four studies contributed to this review finding. There were no or minor concerns regarding methodological limitations, relevance coherence and adequacy. |
| **Available Support and coping means** | | | | | | | |
| **Self-reliance/motivation** | [37-39] | **No or very minor concerns**  There is a concern regarding the reflexivity of Dei-Anane et al. [37] and Gardner et al. [38] (the relationship between the researcher and the participants). However, the credibility of the findings was not impacted by the lack of information about the researcher's role and relationship with the participants. Hence, the review's supporting evidence was evaluated as having minimal or no concerns regarding methodological limitations. | **No or very minor concerns**  One of the three studies that contributed to this finding did not directly focus on the perinatal mental health experience of the mothers. The focus is on the perception of Ghanaian mothers towards PND. However, it demonstrated the experiences of these mothers during the period, thus contributing significantly to the review's findings. | **No concerns**  The review finding was directly and unambiguously supported by three studies, and there were no contradictory findings. | **No or minor concerns**  Three studies contributed to this review finding, providing a variety of examples that illustrate and explain the finding. | **High** | Three studies contributed to this review finding. There were no or minor concerns regarding methodological limitations, relevance coherence and adequacy |
| **Family Network** | [37-38] | **No or very minor concerns**  There is a concern regarding the reflexivity of Dei-Anane et al. [37] and Gardner et al. [38] (the relationship between the researcher and the participants). However, the credibility of the findings was not impacted by the lack of information about the researcher's role and relationship with the participants. Hence, the review's supporting evidence was evaluated as having minimal or no concerns regarding methodological limitations. | **No or minor concerns**  One of the two studies that contributed to this finding did not directly study the perinatal mental health experience of the mothers. Instead, it focused on the perceptions of Ghanaian mothers towards Postnatal Depression (PND). However, it still provided insight into the experiences of these mothers during that period and contributed significantly to the review's findings. | **No concerns**  The two studies fit clearly and coherently into the review's findings. | **Moderate concerns**  Two studies contributed to this review, providing examples that illustrate and explain the finding. We concluded that we have moderate concerns about the data adequacy. | **Moderate** | Two studies contributed to the finding. There are no concerns for the methodological limitation, relevance and coherence but moderate concern for the adequacy. |
| **Faith** | [38] | **No or very minor concerns**  There is a concern regarding the reflexivity of Gardner et al. [38] (the relationship between the researcher and the participants). However, the credibility of the findings was not impacted by the lack of information about the researcher's role and relationship with the participants. Hence, the review's supporting evidence was evaluated as having minimal or no concerns regarding methodological limitations. | **No concerns**  The only study contributing to this finding is of direct relevance to all the dimensions of context. Hence, there are no concerns regarding relevance. | **No concerns**  Although there was only one study contributing to this finding, it was clear and coherent enough to be included in the review without any contradictory results. | **Moderate concerns**  Only one of the four studies included in this review gave this finding but in more detailed and specific information regarding the factor. Based on the overall richness and quantity of the data, we concluded that we only have moderate concerns about the data adequacy | **Moderate** | Only a study contributed to the finding. There are no concerns for the methodological limitation, relevance and coherence but moderate concern for the adequacy. |
| **Met and unmet needs and preferences** | [38,39] | **No or very minor concerns**  There is a concern regarding the reflexivity of Gardner et al. [38] (the relationship between the researcher and the participants). However, the credibility of the findings was not impacted by the lack of information about the researcher's role and relationship with the participants. Hence, the review's supporting evidence was evaluated as having minimal or no concerns regarding methodological limitations. | **No concerns**  The dimensions of the context of the studies contributing to this finding are all of direct relevance to the findings. Thus, there are no concerns regarding the relevance. | **No or minor concerns**  Although the core finding was directly supported by two studies one of the studies did not explore it in detail. However, there were no contradictory data. | **Moderate concerns**  The findings were based on two studies, but only one provided detailed information. The other study contributed partially to the findings, leading to moderate concern about the adequacy of the data. | **Moderate** | Two studies contributed to the finding. There are no concerns for the methodological limitation, relevance and coherence but moderate concern for the adequacy. |

37. Dei-Anane E, Poku AA, Boateng S, Poku KO, Amankwa E, Adasa AN, et al. Perceptions of Ghanaian migrant mothers living in London towards postnatal depression during postnatal periods. Am J Geogr Res Rev. 2018;2:1-8. doi: 10.28933/ajgrr-2018-02-0501.

38. Gardner PL, Bunton P, Edge D, Wittkowski A. The experience of postnatal depression in West African mothers living in the United Kingdom: a qualitative study. Midwifery. 2014;30(6):756-63. doi: 10.1016/j.midw.2013.08.001. PubMed PMID: 24016554.

39. Ling L, Eraso Y, Mascio VD. First-generation Nigerian mothers living in the UK and their experience of postnatal depression: an interpretative phenomenological analysis. Ethn Health. 2022;.doi: 10.1080/13557858.2022.

40. Babatunde T, Moreno-Leguizamon CJ. Daily and cultural issues of postnatal depression in African women immigrants in South East London: tips for health professionals. Nur Res Pract. 2012;2012:181640. doi: 10.1155/2012/181640. PubMed PMID: 23056936.
